# Optimization of In Vitro Th17 Polarization for Adoptive Cell Therapy in Chronic Lymphocytic Leukemia

**DOI:** 10.3390/ijms25126324

**Published:** 2024-06-07

**Authors:** Wael Gamal, Melanie Mediavilla-Varela, Angimar Uriepero-Palma, Javier Pinilla-Ibarz, Eva Sahakian

**Affiliations:** 1 Department of Immunology, H. Lee Moffitt Cancer Center & Research Institute, Tampa, FL 33612, USA; 2 Department of Molecular Medicine, Morsani College of Medicine, University of South Florida, Tampa, FL 33612, USA; 3 Department of Malignant Hematology, H. Lee Moffitt Cancer Center & Research Institute, Tampa, FL 33612, USA

**Keywords:** Th17, CLL, adoptive cell therapy, CAR T cell

## Abstract

Although preclinical investigations have shown notable efficacy in solid tumor models utilizing in vitro-differentiated Th17 cells for adoptive cell therapy (ACT), the potential benefits of this strategy in enhancing ACT efficacy in hematological malignancies, such as chronic lymphocytic leukemia (CLL), remain unexplored. CLL is a B-cell malignancy with a clinical challenge of increased resistance to targeted therapies. T-cell therapies, including chimeric antigen receptor (CAR) T cells, have demonstrated limited success in CLL, which is attributed to CLL-mediated T-cell dysfunction and skewing toward immunosuppressive phenotypes. Herein, we illustrate the feasibility of polarizing CD4^+^ T cells from the Eμ-TCL1 murine model, the most representative model for human CLL, into Th17 phenotype, employing a protocol of T-cell activation through the inducible co-stimulator (ICOS) alongside a polarizing cytokine mixture. We demonstrate augmented memory properties of in vitro-polarized IL-17-producing T cells, and preliminary in vivo persistence in leukemia-bearing mice. Our findings gain translational relevance through successful viral transduction of Eμ-TCL1 CD4^+^ T cells with a CD19-targeted CAR construct during in vitro Th17 polarization. Th17 CAR T cells exhibited remarkable persistence upon encountering antigen-expressing target cells. This study represents the first demonstration of the potential of in vitro-differentiated Th17 cells to enhance ACT efficacy in CLL.

## 1. Introduction

T helper 17 (Th17) cells were initially recognized in 2005 as a distinct Th cell lineage separate from Th1 and Th2 cells [1,2]. They play a crucial role in preserving immune balance and safeguarding the host against extracellular pathogens by fostering inflammatory responses [3,4]. While the role of Th17 cells in promoting autoimmune diseases is well established [5,6,7], their involvement in cancer development remains a subject of debate [8,9,10,11]. However, in the context of cancer adoptive cell therapy (ACT), Th17 cells have been previously characterized as a CD4^+^ T-cell subtype with high plasticity and stem cell-like properties [12,13,14]. This is of special importance since the stemness and memory properties of T cells are key for cell survival post-ACT product infusion and for generating durable anti-tumor responses. In murine models, studies have demonstrated that ex vivo-polarized Th17 cells, particularly tyrosinase-related protein 1 (TRP-1)-specific ones, exhibit potent anti-melanoma activity, surpassing that of Th1 cells [12,15]. These effects were primarily due to the in vivo persistence of Th17 cells and their ability to transition into a distinct Th17 subset with Th1-like behaviors and heightened interferon gamma (IFNγ) production post-ACT. Moreover, compared to Th1 cells, in vitro-expanded TRP-1^+^ Th17 cells have shown increased resistance to apoptosis and reduced expression of exhaustion markers [15]. Human chimeric antigen receptor-positive (CAR^+^) Th17 cells were previously expanded ex vivo and have also retained their antitumor effects against mesothelioma [15]. The inducible co-stimulator (ICOS) has been found to improve the polarization and antitumor function of Th17 cells [16,17]. Polarized human Th17 cells redirected with ICOS-based CAR exhibited improved in vivo persistence in mice with established tumors compared to CD28- or 4-1BB-based CAR T cells [18]. Furthermore, the prolonged in vivo persistence of CD8^+^ T cells expressing CD28- or 4-1BB-based CAR relied on co-infusion with CD4^+^ T cells transduced with ICOS-based CAR [19]. The discoveries have driven progress in developing third-generation CAR constructs incorporating either ICOS/4-1BB [19] or ICOS/OX40 [20] co-stimulation domains. This has led to significant improvements in both anti-tumor effectiveness and in vivo persistence.

Chronic lymphocytic leukemia (CLL), a clonal B-cell malignancy, stands as a predominant form of leukemia in Western societies [21]. While the treatment landscape for CLL has notably advanced with the introduction of targeted therapies such as Bruton tyrosine kinase (BTK) and B-cell lymphoma 2 (Bcl2) inhibitors [22,23], achieving a cure remains elusive due to frequent drug resistance and adverse events in patients. ACT, including CAR T cells, has demonstrated remarkable success in various hematological malignancies [24]. However, its efficacy in CLL, with a 20% complete response rate [25], is limited by acquired dysfunction within the T-cell compartment. In CLL, T cells undergo skewing towards a terminally differentiated state, displaying markers of exhaustion, restricted proliferation, impaired cytotoxicity, and diminished ability to form functional immune synapses with CLL B cells [26,27,28]. This compromised T-cell response significantly contributes to the increased susceptibility to secondary malignancies and infections, thereby escalating morbidity and mortality in this disease [29,30]. Hence, there is a critical necessity to investigate innovative approaches to enhance T-cell-based ACT in CLL.

Clinical evidence in CLL suggests an increase in Th17 cell frequencies and/or IL-17 cytokine levels in patients’ peripheral blood, purportedly associated with improved clinical outcomes [31,32,33]. Nevertheless, the precise mechanisms behind this association remain unclear. Additionally, an augmented STAT3/IL-6 signature, encompassing heightened IL-17 production by CAR T cells, correlated with a complete response in patients undergoing CD19 CAR T-cell therapy [34]. Yet, as of now, there is a significant lack of thorough investigation into the potential benefits of utilizing Th17 cells to enhance ACT in CLL, despite the promising clinical prospects that such an approach may offer. In this study, we utilize the Eµ-TCL1 murine model, which is widely recognized as the most established pre-clinical model for CLL, to present initial evidence regarding the feasibility of inducing a Th17 phenotype in CLL-exposed CD4^+^ T cells through the application and assessment of a protocol involving anti-CD3/anti-ICOS bead formulation. Additionally, we explore the therapeutic potential of generating and evaluating CAR Th17 cells using CLL CD4^+^ T cells.

## 2. Results

### 2.1. Optimization of ICOS-Based Co-Stimulation for In Vitro Th17 Polarization

**Figure 1:**
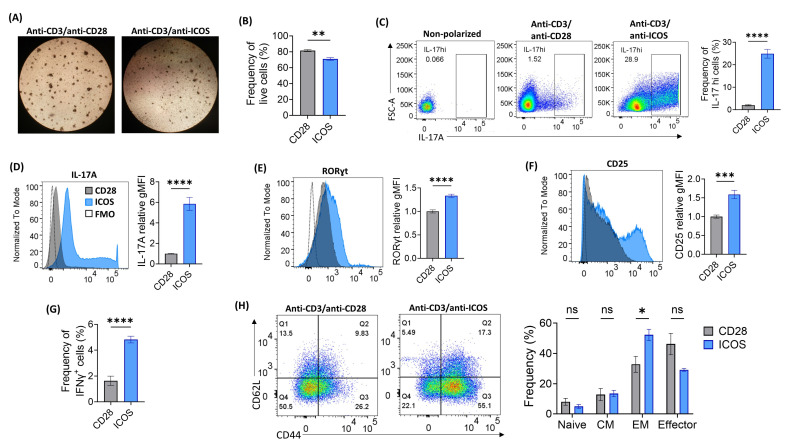
Optimization of ICOS-based co-stimulation for in vitro Th17 polarization. Bulk CD4^+^ T cells from wild-type (WT) spleens were activated using anti-CD28 or anti-ICOS-based dynabeads for 5 days under Th17 polarizing conditions, then subjected to 4–5 h PMA/ionomycin stimulation followed by flow cytometry staining. (**A**) Light microscopy images at 4× magnification comparing T-cell activation clusters at day 5 using the different activation conditions. (**B**) Frequency of live cells as indicated by near-IR fluorescent reactive dye using flow cytometry. (**C**) Representative flow cytometry dot plots and tabulated results for IL-17A production using the different activation beads. (**D**) Quantification of relative IL-17A MFI (mean fluorescence intensity). (**E**,**F**) Representative flow cytometry histograms and tabulated results for intracellular RORγt (**E**) and surface CD25 (**F**) expression levels relative to control (anti-CD28 activation condition). (**G**) Frequency of IFNγ positive cells as indicated by flow cytometry. (**H**) Representative dot plots and tabulated results of T-cell memory phenotypes (naïve: CD44^−^CD62L^+^, central memory (CM): CD44^+^CD62L^+^, effector memory (EM): CD44^+^CD62L^−^ and effector: CD44^−^CD62L^−^). Data presented as mean ± SEM and differences analyzed using paired Student’s *t*-test (* *p* < 0.05; ** *p* < 0.01; *** *p* < 0.001; **** *p* < 0.0001; ns, non-significant).

To establish a procedure for in vitro polarization of Th17 cells, we began by refining a previously published protocol employing ICOS-based stimulation and polarization of Trp-1-specific T cells [16]. Our protocol modifications involved optimizing the concentration of Th17 polarizing cytokines, the composition and ratio of activation dynabeads, and the duration of cell culture. Our formulated anti-CD3/anti-ICOS beads effectively stimulated wild-type (WT) CD4^+^ T cells, as evidenced by the formation of cell clusters observed under light microscopy, with approximately 70% cell viability (Figure 1A,B). However, ICOS-stimulated cells showed slower proliferative capacity, particularly under polarized conditions, compared to their CD28-stimulated counterparts, with lower PD-1 expression (Figure S1A–C). Notably, stimulation with anti-CD3/anti-ICOS beads significantly augmented the production of IL-17A compared to stimulation with anti-CD3/anti-CD28 following polarization with a Th17-inducing cytokine mixture (Table 1 and Figure 1C,D). Furthermore, ICOS-mediated stimulation led to increased expression of the Th17 key transcription factor (TF) retinoic acid-related orphan receptor gamma t (RORγt) (Figure 1E) and elevated CD25 surface expression, which was associated with the remarkable increase in IL-17A production in these cells (Figure 1F and Figure S2A). The enrichment of the Th17-cell signature during polarization was progressive, as indicated by sequential time points extending up to 5 days (Figure S2B). Intracellular staining demonstrated heightened IFNγ production from ICOS-stimulated cells compared to their CD28-stimulated counterparts (Figure 1G). Furthermore, ICOS-based Th17 polarization significantly increased effector memory differentiation of CD4^+^ T cells, while decreasing terminal effector cells (Figure 1H).

Th17 cells in humans are distinguished by their concurrent expression of chemokine receptors, CCR4 and CCR6. We confirmed the efficacy of the refined Th17 polarization protocol utilizing ICOS beads to stimulate FACS-sorted CD4^+^ CCR4^+^ CCR6^+^ cells from healthy donors (Figure S3A). These cells were successfully polarized towards IL-17A production compared to stimulation with anti-CD3/anti-CD28 (Figure S3B). Consistent with observations in murine cells, Th17 cells polarized with ICOS exhibited elevated levels of IFNγ as well as reduced levels of CD95, indicating decreased apoptosis (Figure S3C,D).

### 2.2. ICOS Co-Stimulation Effectively Polarizes Eµ-TCL1 CD4^+^ T Cells into Th17 Phenotype

**Figure 2:**
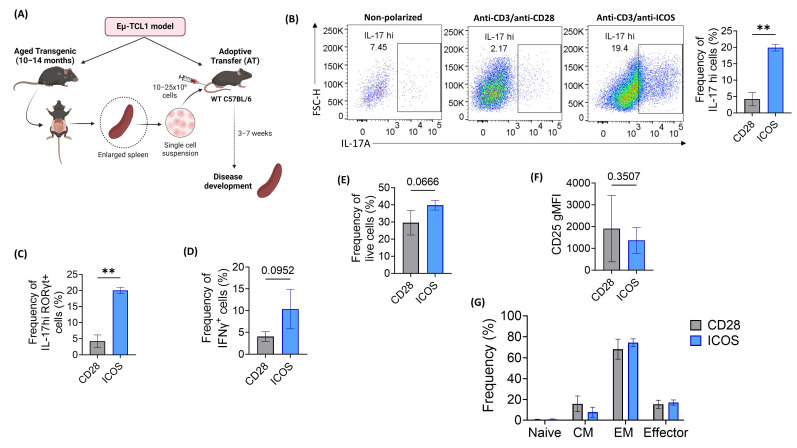
ICOS-based co-stimulation enhances in vitro Th17 polarization of adoptive transfer (AT) Eµ-TCL1 CD4^+^ T cells. Bulk CD4^+^ T cells were negatively selected from AT Eµ-TCL1 splenocytes and polarized to Th17 phenotype using either anti-CD28 or anti-ICOS dynabeads for 5 days. Cells were then stimulated using PMA/ionomycin and stained for flow cytometry. (**A**) Diagram representing the establishment of the AT Eµ-TCL1 model. (**B**) Representative flow cytometry dot plots and tabulated results of IL-17A production by polarized CD4^+^ T cells. (**C**) Frequency of IL-17^hi^ RORγt^+^ cells as measured by intracellular staining. (**D**,**E**) Frequency of IFNγ positive cells (**D**) and live cells (**E**) as indicated by flow cytometry analysis. (**F**) Surface expression levels of CD25. (**G**) Analysis of T-cell memory phenotypes (naïve: CD44^−^CD62L^+^, central memory (CM): CD44^+^CD62L^+^, effector memory (EM): CD44^+^CD62L^−^ and effector: CD44^−^CD62L^−^). Data were presented as mean ± SD and differences were analyzed using paired Student’s *t*-test (** *p* < 0.01).

Previous studies have demonstrated a shift in the phenotype of CD4^+^ Th cells in CLL [27,33,35,36,37,38]. We demonstrated this alteration by conducting a cytokine analysis, revealing a significant increase in IL-10, IFNγ, tumor necrosis factor alpha (TNFα), and IL-17 from transgenic Eµ-TCL1 CD4^+^ T cells following in vitro activation using anti-CD3/anti-CD28 (Figure S4A). Building on these findings, we investigated whether we could effectively polarize the skewed Eµ-TCL1 CD4^+^ T cells into Th17 cells in vitro using our optimized protocol. We utilized the AT Eµ-TCL1 murine model to address the latency and heterogenous disease development in the transgenic Eµ-TCL1 mice. This model was established by adoptively transferring Eμ-TCL1 leukemic cells into WT C57BL/6 mice (Figure 2A). Indeed, ICOS-based Th17 polarization significantly enhanced the Th17 signature (IL-17A and RORγt production) by AT Eµ-TCL1 CD4^+^ T cells compared to CD28 stimulation (Figure 2B,C). Following in vitro stimulation, ICOS-stimulated cells showed an increase in IFNγ production, accompanied by a slight improvement in cell viability (Figure 2D,E). However, we did not observe a notable change in CD25 expression following ICOS stimulation (Figure 2F). Furthermore, ICOS-stimulated cells exhibited an increase in effector memory profile, which is consistent with observations in WT T cells (Figure 2G).

Furthermore, we successfully confirmed the capability of CD4^+^ T cells isolated from aged transgenic Eµ-TCL1 mice to polarize towards the Th17 phenotype using the ICOS-based polarization protocol (Figure S4B,C). Like AT Eµ-TCL1 cells, we did not detect a significant change in CD25 expression following ICOS-based polarization of cells from transgenic mice (Figure S4D). Additionally, we validated the increase in effector memory and decrease in terminal effector cells through ICOS-based polarization of cells from transgenic mice (Figure S4E). Cytokine analysis of culture media revealed an increase in IL-17A, IL-6, and IFNγ production from ICOS-polarized cells compared to CD28-polarized counterparts (Figure S4F).

### 2.3. Eµ-TCL1 In Vitro-Polarized Th17 Cells Show Signs of Enhanced Memory Formation and In Vivo Persistence

**Figure 3:**
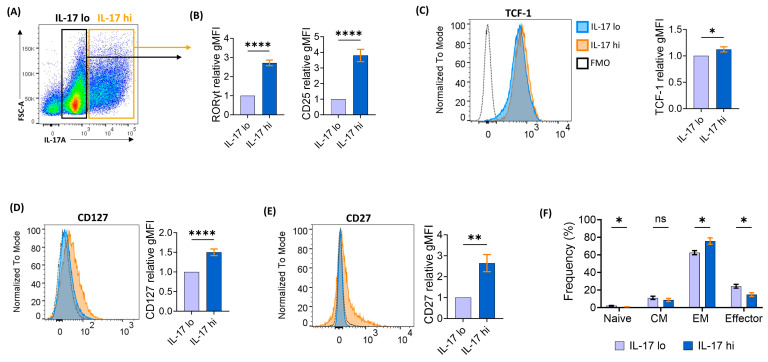
Eµ-TCL1 in vitro-polarized Th17 cells show signs of enhanced memory formation. (**A**) Representative flow cytometry dot plot showing gating on Eµ-TCL1 CD4^+^ T-cell populations with varying levels of IL-17 expression following in vitro Th17 polarization for 5 days. (**B**–**E**) Relative expression levels of RORγt, CD25 (**B**), TCF-1 (**C**), CD127 (**D**), and CD27 (**E**) in IL-17^hi^ populations compared to IL-17^lo^ counterparts. (**F**) Analysis of T-cell memory phenotypes (naïve: CD44^-^CD62L^+^, central memory (CM): CD44^+^CD62L^+^, effector memory (EM): CD44^+^CD62L^−^ and effector: CD44^−^CD62L^−^) in IL-17^hi^ and IL-17^lo^ cell populations. Data presented as mean ± SEM and differences analyzed using unpaired Student’s *t*-test (* *p* < 0.05; ** *p* < 0.01; **** *p* < 0.0001; ns, non-significant).

To delve deeper into the memory characteristics of Th17 polarized Eµ-TCL1 CD4^+^ T cells, we examined cells expressing high levels of IL-17A (IL-17^hi^) versus those expressing low levels (IL-17^lo^) (Figure 3A,B). IL-17^hi^ cells were more activated, as indicated by higher CD25 expression (Figure 3B), and they exhibited a significant upregulation of the self-renewal transcription factor TCF-1 (Figure 3C). In addition, they displayed increased expression of the IL-7 receptor (CD127) and the memory marker CD27 (Figure 3D,E). Notably, IL-17^hi^ cells predominantly displayed an effector memory phenotype compared to IL-17^lo^ cells (Figure 3F). Our preliminary results of infusing in vitro-polarized Eµ-TCL1 Th17 cells into CLL-bearing mice (Figure S5A) showed that the infused circulating Th17 cells maintain a capacity to produce both IL-17 and IFNγ when in vitro-stimulated with PMA/ionomycin 4 days after injection into mice (Figure S5B). Significantly, these infused Th17 cells showed remarkable persistence in both the peripheral blood (up to 41 days) and also in lymphoid organs when analyzed 49 days post-infusion (Figure S5C,D). Additionally, these preliminary findings demonstrated a slower leukemia progression and improved mice survival post-infusion of Th17 cells (Figure S5E,F). These findings underscore the potential benefit of utilizing Th17-polarized cells as a persistent T-cell phenotype for ACT in CLL.

### 2.4. Eµ-TCL1 CAR Th17 Cells Maintain In Vitro Cytotoxic Potential with Improved Persistence

**Figure 4:**
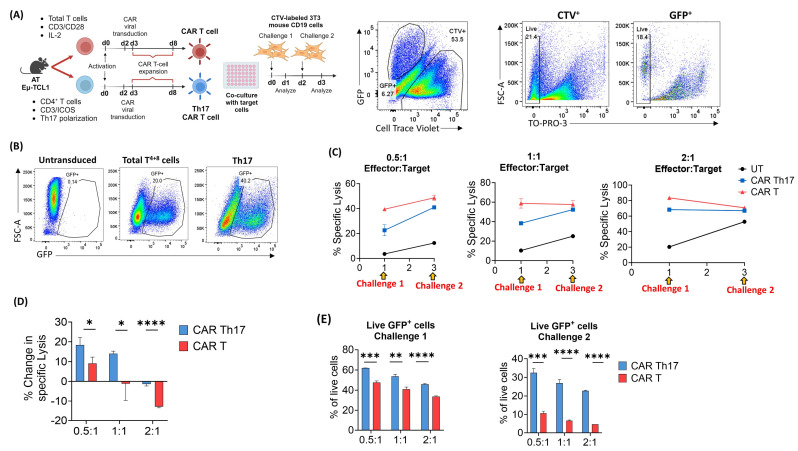
Eµ-TCL1 CAR Th17 cells maintain in vitro cytotoxic potential with improved persistence. (**A**) Schematic showing the timeline for the in vitro generation, polarization, and challenging of CAR Th17 cells with CD19^+^ 3T3 target cells. Sample flow cytometry dot plots showing gating on live and dead 3T3 and CAR T cells are shown. (**B**) Representative flow cytometry dot plots showing viral transduction efficiency as indicated by percent GFP^+^ CAR T cells. (**C**) Quantitation of percent specific lysis upon two challenges of CD19^+^ 3T3 target cells at different effector/target (CAR T cell: 3T3 cell) ratios. (**D**) Percentage change in target cell lysis by CAR T cells between the two target cell challenges at different effector/target cell ratios. (**E**) Percentage of live GFP^+^ CAR T cells after the first and second challenges with 3T3 target cells. Data presented as mean ± SD and differences analyzed using unpaired Student’s *t*-test (* *p* < 0.05; ** *p* < 0.01; *** *p* < 0.001; **** *p* < 0.0001).

To evaluate the cytotoxic capacity of Eµ-TCL1-derived T cells under Th17 polarization, we employed a CD19-targeted CAR T-cell approach. To enhance the translational relevance of our study, we compared in vitro-polarized Th17 CAR T cells with CAR T cells generated from a total T-cell pool (CD4^+^ and CD8^+^ T cells, referred to as Eµ-TCL1 CAR T^4+8^ cells) (Figure 4A), simulating the actual CAR T-cell product manufacturing process for patients. AT Eµ-TCL1 bulk CD4^+^ T cells were polarized to the Th17 phenotype using ICOS-based co-stimulation and transduced 48 h later with retroviral vectors carrying the CD19 CAR construct containing the CD28 costimulatory domain, followed by expansion in Th17 polarizing conditions (Figure 4A). Concurrently, total T cells isolated from the same AT Eµ-TCL1 spleens were conventionally activated with anti-CD3/anti-CD28 dynabeads and transduced with the CAR construct without any in vitro polarization. Notably, Th17 CAR T cells showed improved viral transduction efficiency compared to Eµ-TCL1 CAR T^4+8^ cells (Figure 4B); therefore, the CAR^+^ (GFP^+^) percentage was taken into consideration while seeding the number of required cells for the cytotoxicity assay. Eµ-TCL1 CAR Th17 cells showed a distinct IL-17^+^ RORγt^+^ population compared to CAR T^4+8^ cells (Figure S6A), with the capacity to produce IFNγ, albeit to a lower extent compared to the CAR T^4+8^ cells (Figure S6B). Seven days post the initial activation, Eµ-TCL1 CAR T^4+8^ cells were mainly composed of CD8^+^ (non-CD4^+^) CAR T cells with only a residual population of CD4^+^ T cells (Figure S6C). The in vitro cytotoxic potential of the generated CAR Th17 cells was compared to the conventional Eµ-TCL1 CAR T^4+8^ cells using two challenges of cell trace violet-labeled 3T3 cells expressing mouse CD19 target antigen (Figure 4C). The identification of live and dead cells was performed using the TO-PRO-3 flow cytometry stain. After the first challenge, CAR Th17 cells exhibited a noteworthy ability to eliminate target cells across various effector cell-to-target ratios, compared to untransduced controls; however, it was less than that of CAR T^4+8^ cells (Figure 4C). Following the second challenge with target cells, CAR Th17 cells exhibited an enhanced specific killing capacity, rendering them comparable to the Eµ-TCL1 CAR T^4+8^ cells (Figure 4C). It is significant to note that, compared to the first challenge, Eµ-TCL1 CAR Th17 cells showed a marked enhancement in the specific lysis of target cells during the second challenge. In contrast, Eµ-TCL1 CAR T^4+8^ cells exhibited a net decrease in their killing capacity at 1:1 and 2:1 effector-to-target ratios (Figure 4D). Interestingly, Eµ-TCL1 CAR^+^ Th17 cells exhibited a remarkable improvement in cell viability compared to Eµ-TCL1 CAR T^4+8^ cells (Figure 4E), confirming the persistence capacity of Th17 cells post-target challenging.

## 3. Discussion

Autologous T-cell therapies, incorporating CD8^+^ and CD4^+^ CAR-expressing T cells, demonstrate efficacy across multiple hematological malignancies, such as diffuse large B-cell lymphoma (DLBCL) and acute lymphocytic leukemia (ALL) [39,40]. Historically, CD8^+^ T cells have been ascribed a primary effector role within ACT products, with CD4^+^ T cells traditionally presumed to provide supportive functions. Recent evidence, however, indicates that the CD4^+^ T-cell subset within these products exhibits cytolytic activity. Recently, Boulch et al. demonstrated the significant role of IFNγ as a key determinant in enhancing the efficacy of CD4^+^ CAR T cells through both the direct and indirect killing of B-cell tumors [41]. Using the syngeneic murine model, CD4^+^ CAR T cells were enough to eliminate ALL without the presence of CD8^+^ T cells [42]. Furthermore, a recent investigation revealed that a limited population of CD4^+^ T cells, by engaging with antigen-presenting cells, can eliminate interferon-unresponsive and major histocompatibility complex (MHC)-deficient tumors that evade direct targeting by CD8^+^ T cells [43]. Clinically, a lasting response was seen in a metastatic melanoma patient who received treatment with an autologous HLA-DP4-restricted NY-ESO-1-specific CD4^+^ T-cell clone [44]. Another clinical trial on cancer patients demonstrated the efficacy and safety of CD4^+^ T cells genetically engineered to target the MAGE-A3 antigen [45]. Similarly, another patient with metastatic cholangiocarcinoma exhibited an effective response when treated with CD4^+^ T cells reactive to mutated ERBB2IP, which were cultivated from tumor-infiltrating lymphocytes [46].

Indeed, the focus on the significance of the CD4^+^ T-cell component in CAR T-cell products [47,48,49] has resulted in the FDA approval of lisocabtagene maraleucel (liso-cel), a CAR T-cell infusion product containing a 1:1 ratio of CD4^+^ and CD8^+^ CAR T cells infused separately for treating large B-cell lymphoma (LBCL). These findings align with evidence indicating reduced exhaustion of CD4^+^ CAR T cells [42,50] and their persistence for over a decade in two CLL patients who were treated with CAR T-cell therapy [51]. Despite that, further studies are required to elucidate the potential benefits of employing solely CD4^+^ T-cell ACT products or augmenting CD8^+^ T cells with particular phenotypes of CD4^+^ T cells to enhance the durability and effectiveness of ACT in hematological malignancies.

Among the diverse CD4^+^ T-cell phenotypes, Th17 cells have been recognized for their attributes favoring prolonged persistence and self-renewal following ACT [12,14,52]. This persistence is associated with remarkable anti-tumor activity, particularly through the polarization of Th17 cells into IFNγ-producing Th17 cells in vivo [12,16,17,52]. Studies have also shown success in generating Th17 CAR T cells targeting mesothelioma, which demonstrated efficacy in a xenograft murine model [17,53]. Despite these advancements, the exploration of the feasibility and efficacy of utilizing CD4^+^ CAR T cells, especially those with a Th17 phenotype, in the context of CLL has not been explored.

CLL represents a B-cell malignancy characterized by a restricted response to CAR T-cell therapy [25], primarily attributable to the pronounced T-cell dysfunction linked with this condition [26,27,28,34]. One notable expression of this dysfunction is the deviation of CD4^+^ Th cell differentiation in CLL patients and the Eμ-TCL1 murine model towards either Th2 or Th1 phenotypes [27,36,37,54,55,56]. In contrast, multiple studies have also suggested a remarkable abundance of Tregs in the peripheral blood of CLL patients, corresponding to a worse prognosis [57,58,59,60,61]. Th17 cells and/or IL-17 cytokine levels are elevated in the peripheral blood from CLL patients versus healthy donors, which was claimed to be associated with positive clinical outcomes [31,32,33]. Our results using Eμ-TCL1-isolated CD4^+^ T cells confirm the skewing of these cells post in vitro activation as indicated by the elevated levels of IFNγ, TNFα, IL-10, and IL-17A. However, to date, no research has investigated the feasibility of in vitro generating the Th17 phenotype using these skewed CLL T cells to enhance the persistence and efficacy of ACT in CLL. Herein, through the refinement of an already established Th17 polarization protocol [16], we were able to successfully polarize Eμ-TCL1 CD4^+^ T cells into a Th17 phenotype capable of producing high levels of IL-17A and RORγt. The injection of these cells into CLL-bearing mice resulted in successful engraftment, persistence, and maintenance of IL-17 production at least at the initial time point post-infusion. Moreover, the injected cells demonstrated a capacity to produce IFNγ when stimulated in vitro. Additionally, we demonstrate here the improved viral transduction efficiency with CD19 CAR using ICOS co-stimulation and Th17 polarization of Eμ-TCL1 CD4^+^ T cells. Results from the in vitro cytotoxicity assay revealed that the generated Eμ-TCL1 CAR Th17 cells exhibited an enhanced ability to kill target cells upon a subsequent challenge while maintaining higher cell viability. However, while the overall cytotoxic capability of Eμ-TCL1 CAR Th17 cells did not surpass that of Eµ-TCL1 CAR T^4+8^ cells, the prolonged persistence of CAR Th17 cells in culture suggests a potential for enhanced in vivo availability. In this study, we used CAR T cells generated from a total T-cell pool as a control to compare our CAR Th17 product to a real-world example of CAR T cells used in patients. However, based on the results from the current study, it would be interesting to compare ICOS versus CD28-mediated co-stimulation in the generation of CAR Th17 cells from a CLL source in our proposed experiments. Additionally, future plans involve conducting trials where ex vivo polarized Eµ-TCL1 CAR Th17 cells are infused alone or in combination with CD8^+^ CAR T cells into leukemia-bearing mice. Long-term survival and anti-leukemic effects will be monitored and compared with conventional CAR T-cell products generated from a pool of CD8^+^ and CD4^+^ T cells. This work can be translated into clinical studies using CLL patient cells to generate CAR Th17 products that can be tested in xenograft murine models.

## 4. Materials and Methods

### 4.1. Adoptive Transfer (AT) Eμ-TCL1 Murine Model

To initiate CLL, we extracted 10–25 × 10^6^ leukemic splenocytes from aged Eμ-TCL1 transgenic mice and transferred them through adoptive transfer (AT) into female wild-type (WT) syngeneic C57BL/6 mice aged 6–8 weeks via tail vein injection. We categorized aged leukemic Eμ-TCL1 mice as those over 8 months old, exhibiting over 70% CD5^+^ B cells within the lymphocyte gate in peripheral blood (PB) as assessed by flow cytometry. Disease onset in AT mice was confirmed by observing increased counts and proportions of malignant B cells (CD19^+^ CD5^+^ B220^low^) in PB within 3–7 weeks post-transfer. Subsequently, we collected blood samples and spleens from mice for T-cell isolation, immunophenotyping, or tumor burden analysis. Throughout the experiments, we ensured the age and sex matching of mice. All animal protocols were subject to review and approval by the Institutional Animal Care and Use Committee, Research Integrity and Compliance, University of South Florida, Tampa, FL, USA.

### 4.2. Th17 Cell Polarization and Culture

Splenocytes from either WT or Eμ-TCL1 mice were obtained as single-cell suspensions by mechanically homogenizing spleens through a 70 µm cell strainer. Following this, the cells underwent washing and ACK lysis, and they were subsequently utilized for either flow cytometry analysis or T-cell isolation. CD4^+^ T cells were negatively selected from AT Eμ-TCL1 splenocytes using an EasySep CD4^+^ T-cell isolation kit (StemCell, Cambridge, MA, USA, purity routinely > 85%). CD4^+^ T cells were activated using either anti-CD3/anti-ICOS or anti-CD3/anti-CD28 beads (formulated in the lab using M-450 Tosylactivated dynabeads (ThermoFisher, Vilnius, Lithuania) according to the manufacturer’s instructions) in the presence or absence of a Th17 polarizing cytokine mixture (Table 1). Cells were cultured for 5–7 days at 37 °C and 5% CO_2_ in complete RPMI medium (cRPMI): RPMI 1640 with L-glutamine (Corning, Manassas, VA, USA) plus 10% fetal bovine serum (Biowest, Bradenton, FL, USA), 1% penicillin-streptomycin (Gibco/ThermoFisher, Grand Island, NY, USA), 1% nonessential amino acids (Corning), 0.2% MycoZap (Lonza, Walkersville, MD, USA), 55 µM β-mercaptoethanol (Gibco), and 50 µg/mL gentamicin (Gibco). Cells were split to keep a cell density of 1–2 × 10^6^/mL.

### 4.3. ACT Using Polarized Th17 Cells

Female 6–8-week-old CD45.1 C57BL/6 mice were purchased from Charles River, Wilmington, MA, USA. CLL induction was performed as described above using leukemic splenocytes from CD45.2 Eμ-TCL1 mice. After leukemia development, CD45.1 recipient mice were randomized into treatment groups and lymphodepleted with a 250 mg/kg I.P. dose of cyclophosphamide monohydrate (Sigma, St. Louis, MO, USA) one day before ACT. On day 6 of culture, 20 × 10^6^ CD45.2 Eμ-TCL1 CD4^+^ T cells activated with anti-CD3/anti-ICOS beads and polarized to Th17 were intravenously injected into the leukemic CD45.1 recipient mice. Peripheral CLL burden and Th17-cell in vivo persistence were monitored via weekly submandibular bleeds and flow cytometry analysis. Mice were monitored for survival through daily inspections. Mice were euthanized when there was evidence of progressing leukemia, such as reduced activity, hunched posture, or shallow breathing.

### 4.4. Flow Cytometry Staining

Initially, cells were incubated with a viability dye for 30 min at room temperature (RT) to exclude dead cells. Following this, surface staining was performed by incubating the cells with the specified antibodies (Table S1) for 30 min at 4 °C in FACS buffer, consisting of phosphate-buffered saline (PBS) with 2% fetal bovine serum. Subsequently, intracellular staining was conducted on surface-stained cells by fixing and permeabilizing them using the Foxp3 transcription factor buffer set (eBioscience/ThermoFisher, Carlsbad, CA, USA) for 30–60 min at RT, followed by incubation with the designated antibodies (Table S1) for 30–60 min at RT in 1× permeabilization buffer. For in vitro cytokine production analysis, cells were stimulated with Phorbol 12-myristate 13-acetate (PMA, 50 ng/mL, Sigma, St. Louis, MO, USA) and ionomycin (750 ng/mL, Sigma) for 4–5 h, with GolgiStop (1:1500, BD Biosciences, San Diego, CA, USA) added 1-h post-stimulation. Subsequently, cells were stained with antibodies against specific cytokines using the same intracellular staining protocol.

### 4.5. Cell Trace Violet (CTV) Proliferation Assay

CD4^+^ T cells were isolated from either WT or transgenic Eµ-TCL1 mice spleens using EasySep CD4^+^ T-cell isolation kit (StemCell, Cambridge, MA, USA). Cells were labelled with 2 µM CTV stain, stimulated with anti-ICOS or anti-CD28 dynabeads, and then cultured as described above in either Th17 polarized or non-polarized conditions. Percentage divided cells was analyzed by flow cytometry through measuring the dilution of CTV dye, on days 3 and 5 during culture.

### 4.6. Flow Cytometry Analysis

Flow cytometry acquisition was performed using BD LSRII or BD FACSymphony cytometers (BD Biosciences, San Diego, CA, USA) equipped with BD FACSDiva software v9.3. Positive and negative gates were established for each parameter, and data analysis was conducted using FlowJo software v10.8.1 (BD Biosciences). Gating was executed based on fluorescence minus one (FMO) control. To ensure consistency, normalization of mean fluorescence intensity (MFI) across different experiments was conducted concerning controls.

### 4.7. T-Cell Viral Transduction

The GFP-tagged m1928z CAR construct [62] as well as the retroviral packaging and producer cell lines (H29 and Phoenix E) utilized in this study were generously given by Dr. Marco Davila (Roswell Park, Buffalo, NY, USA). Gammaretroviral production and T-cell transduction were conducted using a previously established method [63,64]. In brief, we transfected the retroviral vectors having the CAR construct into 400 × 10^3^ H29 cells using a calcium phosphate transfection kit (ThermoFisher, Eugene, OR, USA). We then collected the retroviral supernatants from the transfected cells and used them to transduce Phoenix E producer cells. The retroviral supernatants from the transduced Phoenix E cells were then harvested, filtered, and stored at −80 °C for future use. T cells or CD4^+^ T cells were negatively isolated from AT Eμ-TCL1 splenocytes using EasySep mouse T-cell or CD4^+^ T-cell isolation kits, respectively (StemCell). Total T cells were activated using anti-CD3/anti-CD28 dynabeads and 60 IU/mL hIL-2, while CD4^+^ T cells were activated using formulated anti-CD3/anti-ICOS beads and polarized to Th17 as described above. On day 2 post activation, cells were collected, washed, and re-seeded at 1 × 10^6^/mL in retronectin (Takara Bio, San Jose, CA, USA)-coated non-tissue culture-treated 6-well plates. Retroviral supernatant was added to the cells, followed by centrifugation for 1 h at 2000× *g* and 32 °C. The following day, we repeated the retroviral spin transduction. Th17 polarization cytokines were replenished every 3 days during the culture and expansion of CAR T cells. CAR T-cell expansion was measured using trypan blue staining and cell counting. Viral transduction efficiency was assessed via flow cytometry, by measuring the percent of GFP-positive cells.

### 4.8. In Vitro Cytotoxicity Analysis

3T3 target cells expressing mouse CD19 were labeled with 2 µM cell trace violet (CTV) and then irradiated. Next, 20 × 10^3^ 3T3 target cells were seeded into 48-well plates and cultured overnight in complete DMEM media (Gibco/ThermoFisher, Grand Island, NY, USA). The following day, media were replaced with cRPMI media, and CAR Th17, CAR total T cells, or untransduced T cells were cocultured with target cells (challenge 1) at either 0.5:1, 1:1, or 2:1 effector/target ratio based on the percentage of GFP^+^ CAR expression. After 24 h, samples were analyzed by flow cytometry to measure the killing capacity of CAR T cells. Then, 48 h after challenge 1, seeded CAR T cells were rechallenged with another 20 × 10^3^ CTV-labelled and irradiated 3T3 target cells (challenge 2). Percent specific killing was measured 24 h later. Percent specific lysis was calculated as [1 − (Live 3T3 cocultured with CAR T/Live 3T3 cultured alone)] × 100.

### 4.9. Statistical Analysis

Analysis of the data was conducted using GraphPad Prism software v10.1.2, and results were presented as mean ± SD or ± SEM as specified in figure captions. Comparisons were assessed using paired or unpaired two-tailed Student’s *t*-tests. Statistical significance was determined at a *p* value < 0.05.

## Figures and Tables

**Table 1:** Cytokine mixture and activation beads used for in vitro Th17 polarization.

**Activation**	**Activation signal**	**Beads:cells ratio**
	CD3/ICOS (M-450 Tosylactivated dynabeads loaded with 50% anti-CD3 and 50% anti-ICOS purified antibodies)	2:1
**Cytokine/Ab mixture**	**Cytokine/Ab**	**Final concentration**
	IL-6	30 ng/mL
	IL-21	100 ng/mL
	IL-1β	10 ng/mL
	TGF-β	3 ng/mL
	Anti-IL-4	10 µg/mL
	Anti-IFNγ	10 µg/mL
	IL-23 (Starting day 3 of culture)	10 ng/mL
	IL-2 (Starting day 3 of culture)	100 IU/mL

## Data Availability

The original contributions presented in the study are included in the article/Supplementary Material, further inquiries can be directed to the corresponding author/s.

## Supplementary Materials

The following supporting information can be downloaded at https://www.mdpi.com/article/10.3390/ijms25126324/s1.
