# Supplementary material for: Optimization of In Vitro Th17 Polarization for Adoptive Cell Therapy in Chronic Lymphocytic Leukemia
Source: Int J Mol Sci. 2024 Jun 7;25(12):6324. doi: 10.3390/ijms25126324 (PMC11203624; doi:10.3390/ijms25126324)
Supplement: Supplementary file 1 [file ijms-25-06324-s001.zip › Supplementary Figure legends 6.05.24.docx]

**Figure S1. ICOS-based co-stimulation results in slower proliferation of CD4^+^ T cells. A,B)** Representative flow cytometry histograms and quantitative results of percentage cell division based on cell trace violet staining of WT or transgenic Eµ-TCL1 CD4^+^ T cells under Th17 polarized **(A)** or non-polarized **(B)** conditions on days 3 and 5 during culture. **C)** Flow cytometric analysis of PD-1 surface expression levels in WT CD4^+^ T cells polarized to Th17 phenotype using anti-ICOS or anti-CD28 stimulation for 3 or 5 days.

**Figure S2. Progressive development of Th17 cells during in vitro culture. A)** Representative flow cytometry dot plots and histograms showing IL-17A expression levels in polarized cells with high CD25 expression. **B)** Tracking Th17 phenotype development, expressing IL-17A and RORɣt, during a 5-days polarization using either ICOS or CD28 dynabeads.

**Figure S3. ICOS-based co-stimulation enhances Th17 polarization from healthy donor (HD) CD4^+^ T cells. A)** Schematic for the process of Th17 polarization using FACS sorted CD4^+^ CCR4^+^ CCR6^+^ HD T cells. **B)** Flow cytometry contour plots showing the increase in IL-17A production using ICOS-based Th17 polarization. **C,D)** Flow cytometry histograms showing the expression levels of IFNɣ **(C)** and CD95 **(D)** using either CD28 or ICOS-based Th17 polarization.

**Figure S4. ICOS-based co-stimulation enhances *ex vivo* Th17 polarization of CD4^+^ T cells from aged transgenic Eµ-TCL1 mice. A)** CBA (cytometric bead assay) flow cytometric analysis of cytokine production from CD4^+^ T cells isolated from aged transgenic Eµ-TCL1 mice splenocytes and expanded for 4-5 days using anti-CD3/anti-CD28 activation and IL-2. **B)** Representative flow cytometry dot plots and tabulated results of IL-17A production by Th17-polarized CD4^+^ T cells from aged transgenic Eµ-TCL1 mice using either anti-CD28 or anti-ICOS-based activation. **C,D)** Frequency of IL-17^+^ RORɣt^+^ cells **(C)** and relative surface expression levels of CD25 **(D)** from anti-CD28 or anti-ICOS-polarized cells. **E)** Analysis of T-cell memory phenotypes (naïve: CD44^-^CD62L^+^, central memory (CM): CD44^+^CD62L^+^, effector memory (EM): CD44^+^CD62L^-^ and effector: CD44^-^CD62L^-^). **F)** CBA analysis of cytokines produced by aged transgenic Eµ-TCL1 CD4^+^ T cells post expansion and Th17 polarization using anti-CD28 or anti-ICOS activation beads. Data presented as mean ± SD **(A,F)** or ± SEM **(B-E)**. Differences analyzed using either unpaired **(A)** or paired **(B-F)** Student's t-test (*p < 0.05; **p < 0.01; ns, non-significant).

**Figure S5. *Ex vivo* ICOS-polarized Eµ-TCL1 Th17 cells show prolonged *in vivo* persistence. A)** Schematic demonstrating the timeline for the procedure of infusing Th7 cells into leukemia-bearing mice. **B)** Representative contour plot showing the dual production of IL-17 and IFNɣ from the *in vivo*- infused Th17 cells after PMA/ionomycin *in vitro* stimulation of peripheral blood sample on day 4 post-infusion. **C)** Representative contour plots and tabulated results showing the percentages of infused Th17 cells in peripheral blood out of CD4^+^ T-cell gate. **D)** Representative contour plots showing percentages of infused Th17 cells in the lymphoid organs of Th17-injected mice 49 days post-infusion. **E)** Quantitation of the absolute cell counts of CLL cells (CD19^+^ B220^lo^ CD5^+^) in the peripheral blood of injected mice. **F)** Kaplan-Meier survival analysis of the different mice groups. N=3-5 mice/group.

**Figure S6. Th17 polarization of Eµ-TCL1 CAR T cells. A-C)** Flow cytometry gating strategy, 5 days after viral transduction with the CD19 CAR construct, showing expression of IL-17/RORɣt **(A)**, IFNɣ **(B)** and CD4 **(C)** in Eµ-TCL1 T cells (CD4^+^ plus CD8^+^) activated with anti-CD3/anti-CD28 or Eµ-TCL1 CD4^+^ T cells activated with anti-CD3/anti-ICOS beads and polarized into Th17.
