# Supplementary material for: Optimization of In Vitro Th17 Polarization for Adoptive Cell Therapy in Chronic Lymphocytic Leukemia
Source: Int J Mol Sci. 2024 Jun 7;25(12):6324. doi: 10.3390/ijms25126324 (PMC11203624; doi:10.3390/ijms25126324)
Supplement: Supplementary file 1 [file ijms-25-06324-s001.zip › Table S1.docx]

**Table S1. List of antibodies and reagents used in this study.**

| **Target**  **Antigen/Reagent** | **Target**  **species**​ | **Fluorochrome**​ | **Clone**​ | **Catalog**  **number**​ | **Manufacturer**​ |
| --- | --- | --- | --- | --- | --- |
| CD4 | Mouse | BUV496 | GK1.5 | 612952 | BD |
| CD19 | Mouse | AF700 | 1D3 | 557958 | BD |
| B220 | Mouse | BV786 | RA3-6B2 | 563894 | BD |
| CD5 | Mouse | BUV737 | 53-7.3 | 612809 | BD |
| CD3e | Mouse | BV421 | 145-2C11 | 562600 | BD |
| PD-1 | Mouse | BV711 | 29F.1A12 | 135231 | Biolegend |
| CD44 | Mouse | AF700 | IM7 | 56-0441-82 | eBioscience​ |
| CD62L | Mouse | BV786 | MEL-14 | 564109 | BD |
| TCF-1 | Mouse | PE-Cy7 | C63D9 | 90511 | Cell Signaling |
| IFNɣ | Mouse | BUV737 | XMG1.2 | 612769 | BD |
| IL-17A | Mouse | PE-CF594 | TC11-18H10 | 562542 | BD |
| RORɣt | Mouse | PE | AFKJS-9 | 12-6988-82 | eBioscience​ |
| CD45.2 | Mouse | AF700 | 104 | 109821 | Biolegend |
| CD25 | Mouse | BUV395 | PC61 | 564022 | BD |
| CD127 | Mouse | BV711 | SB/199 | 565490 | BD |
| CD27 | Mouse | BV421 | LG.3A10 | 124223 | Biolegend |
| TO-PRO-3 Iodide | --- | --- | --- | T3605 | Invitrogen |
| LIVE/DEAD Fixable Near-IR | --- | --- | --- | L34976 | Invitrogen |
| RetroNectin Recombinant Human Fibronectin Fragment | --- | --- | --- | T100B | Takara Bio |
| Dynabeads M-450 Tosylactivated | --- | --- | --- | 14013 | Invitrogen |
| GoInVivo Purified CD278 (ICOS) Antibody, clone | Human/mouse/rat | --- | C398.4A | 313541 | Biolegend |
| Purified Hamster CD3e | Mouse | --- | 145-2C11 | 553058 | BD |
| Purified Hamster CD28 | Mouse | --- | 37.51 | 557393 | BD |
| Dynabeads T-Activator CD3/CD28 | Mouse | --- | --- | 11453D | ThermoFisher |
