# Supplementary figures and images for: Optimization of In Vitro Th17 Polarization for Adoptive Cell Therapy in Chronic Lymphocytic Leukemia
Source: Int J Mol Sci. 2024 Jun 7;25(12):6324. doi: 10.3390/ijms25126324 (PMC11203624; doi:10.3390/ijms25126324)

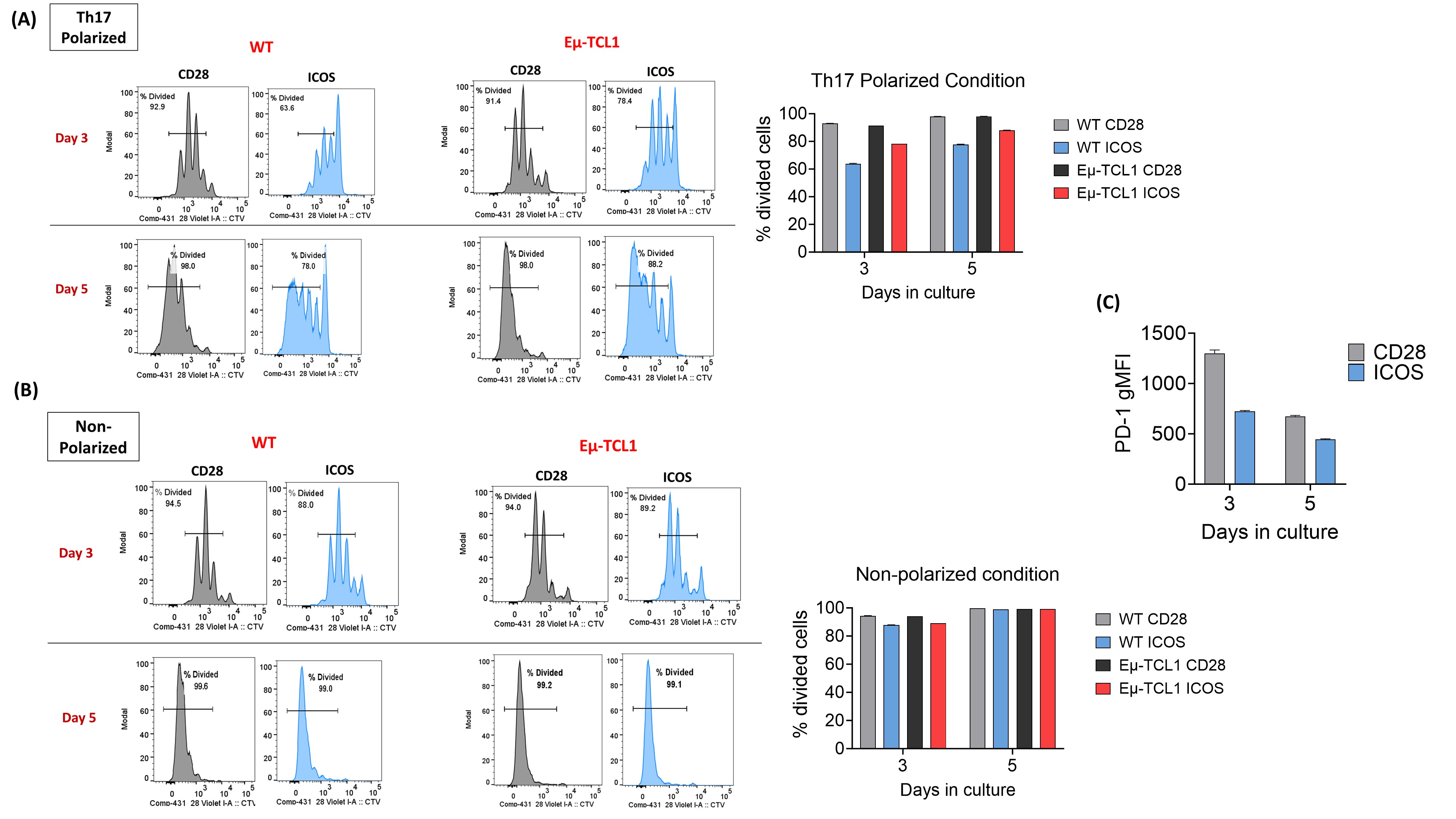

Supplement: Supplementary file 1 [file ijms-25-06324-s001.zip › Fig.S1.JPG]

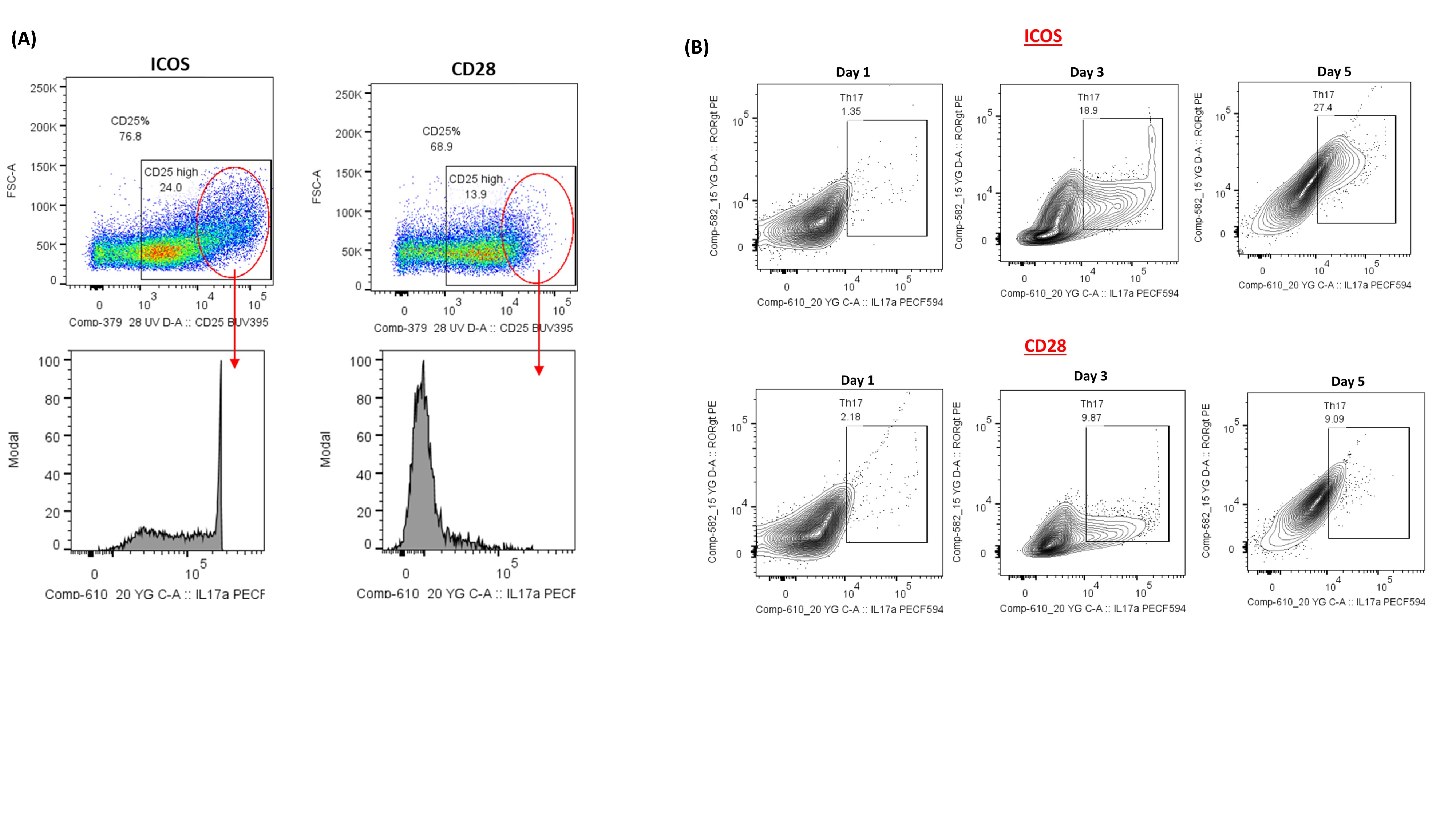

Supplement: Supplementary file 1 [file ijms-25-06324-s001.zip › Fig.S2.JPG]

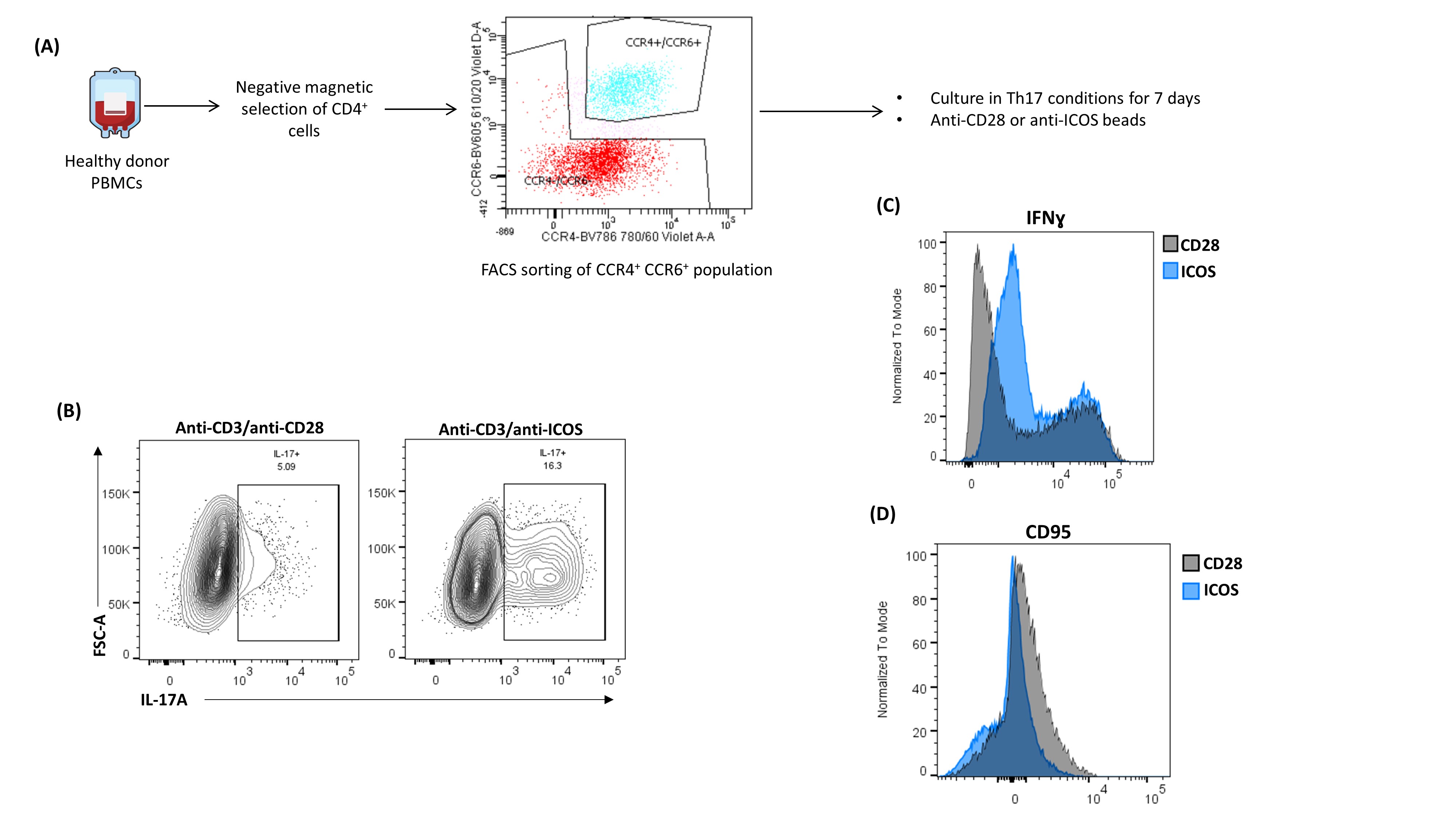

Supplement: Supplementary file 1 [file ijms-25-06324-s001.zip › Fig.S3.JPG]

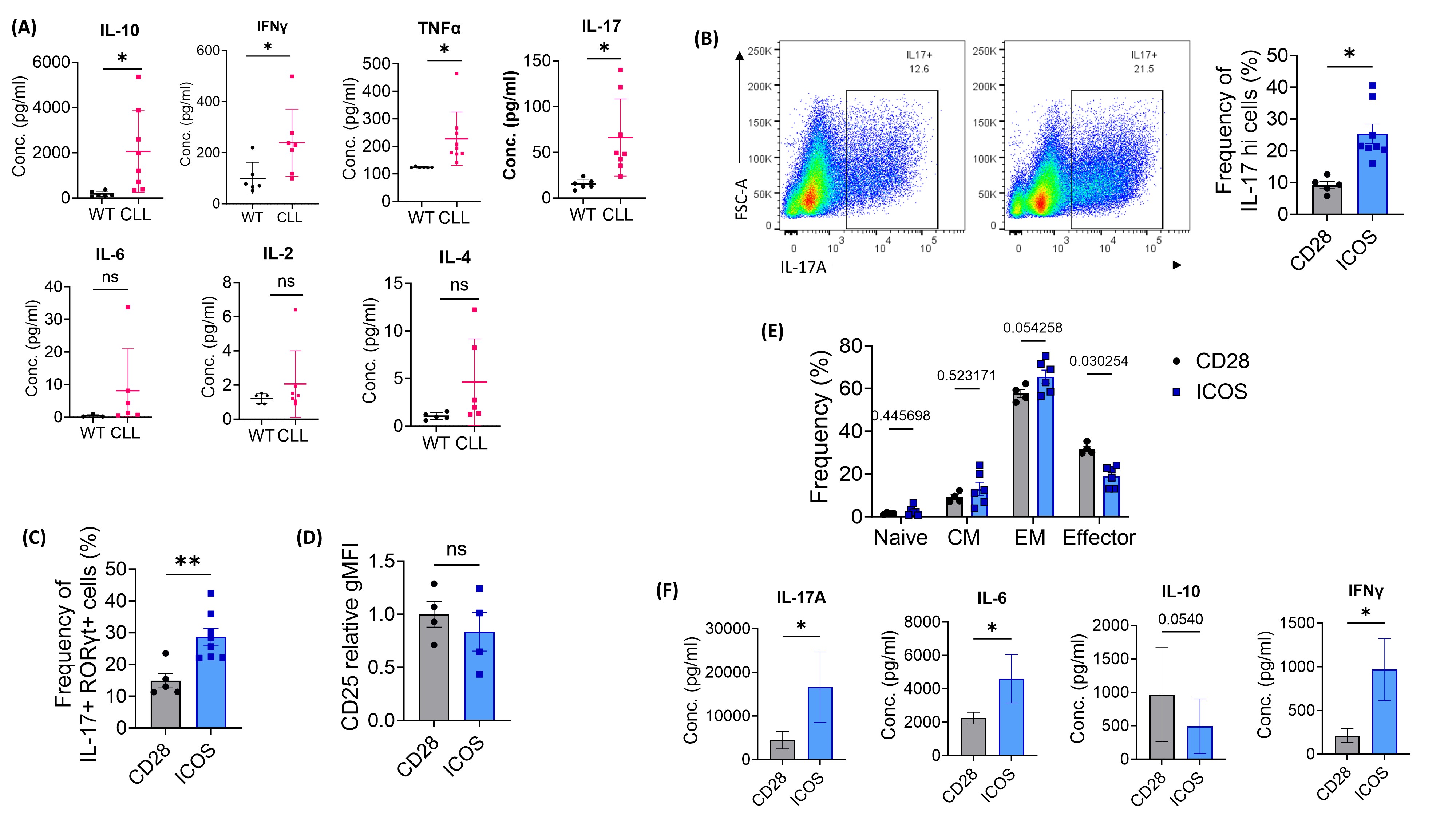

Supplement: Supplementary file 1 [file ijms-25-06324-s001.zip › Fig.S4.JPG]

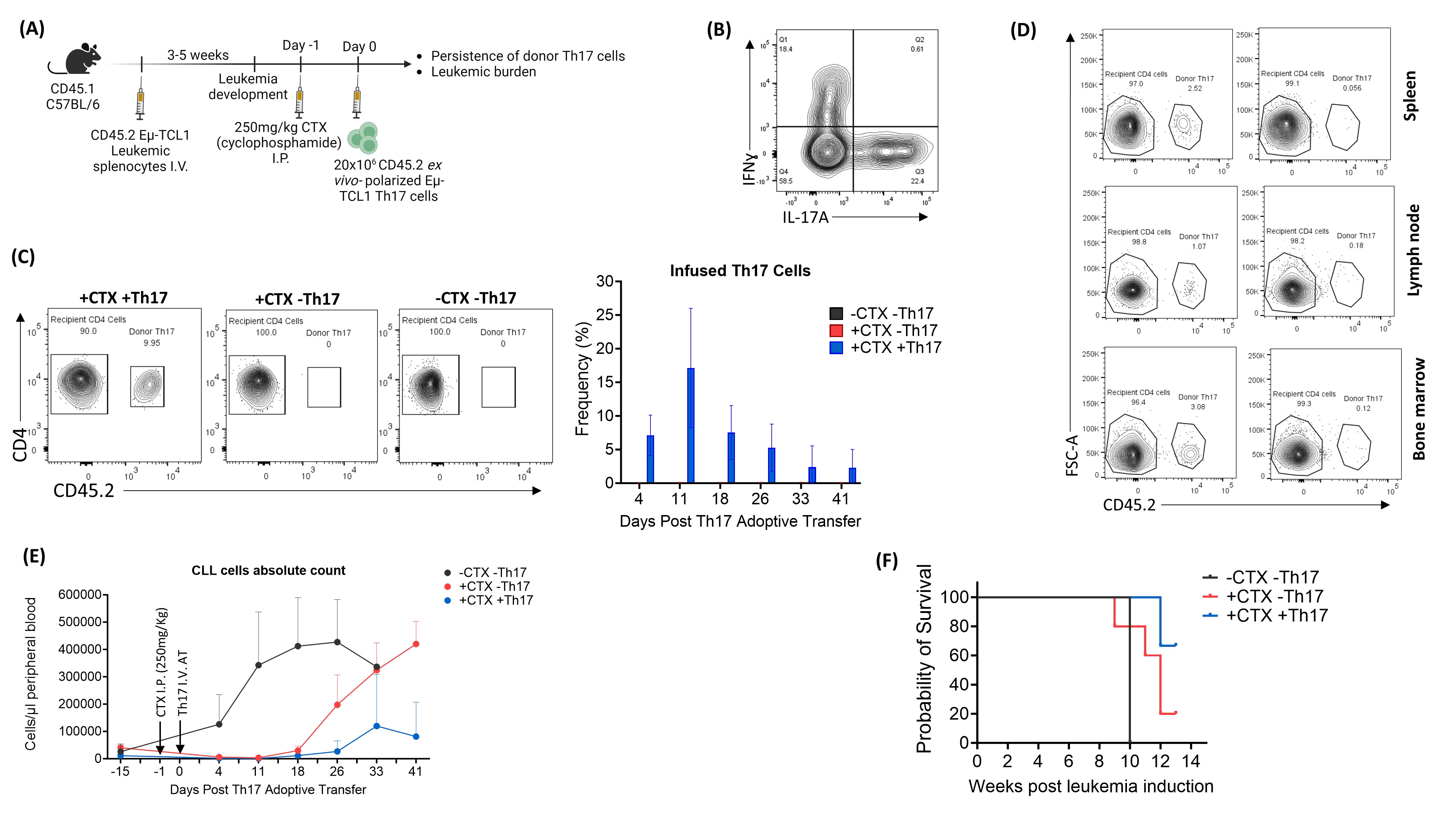

Supplement: Supplementary file 1 [file ijms-25-06324-s001.zip › Fig.S5.JPG]

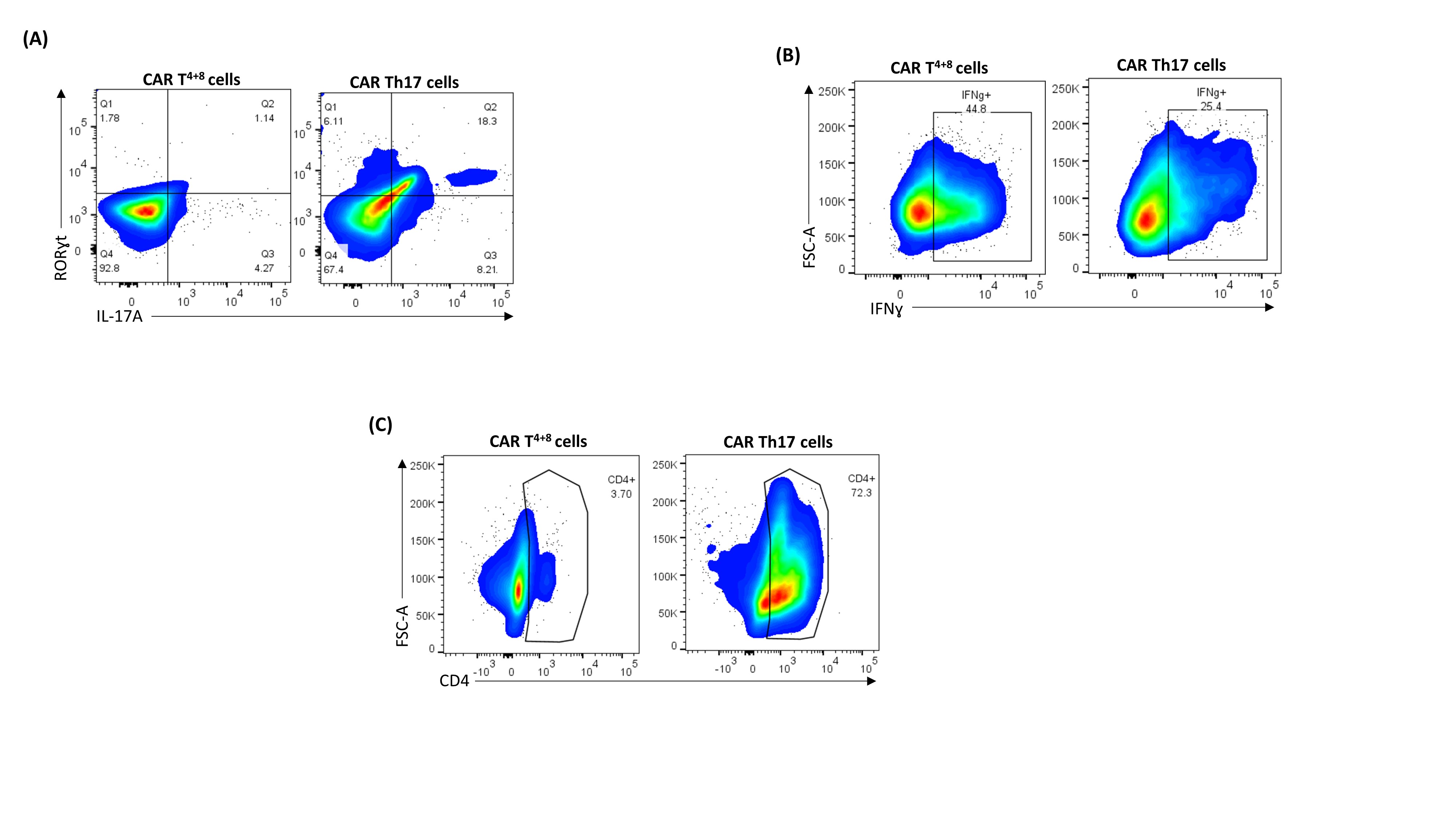

Supplement: Supplementary file 1 [file ijms-25-06324-s001.zip › Fig.S6.JPG]
